# Red Cell Distribution Width-to-Albumin Ratio as an Early Predictor of Intensive Care Requirement and Mortality in Acute Pancreatitis

**DOI:** 10.3390/medicina62020248

**Published:** 2026-01-24

**Authors:** Mehmet Kasım Aydın, Zekiye Nur Harput, Mehmet Cudi Tuncer

**Affiliations:** 1 Department of Gastroenterology, Faculty of Medicine, Mersin University, Mersin 33110, Turkey; zeknurzek@gmail.com; 2 Department of Anatomy, Faculty of Medicine, Dicle University, Diyarbakir 21280, Turkey; drcudi@hotmail.com

**Keywords:** red cell distribution width, albumin, acute pancreatitis, disease severity, prognostic biomarkers

## Abstract

*Background and Objectives*: Acute pancreatitis (AP) is an acute inflammatory disease ranging from mild, self-limiting forms to severe presentations associated with high morbidity and mortality. Early prognostic assessment is crucial for guiding clinical management. This study aimed to evaluate the prognostic value of the red cell distribution width-to-albumin ratio (RDW/Alb, RAR) in relation to clinically relevant outcomes, including intensive care unit (ICU) admission and in-hospital mortality, in patients with AP. *Materials and Methods*: This retrospective study included 282 patients diagnosed with AP who were hospitalized at Mersin University Hospital between January 2019 and February 2024. Clinical, laboratory, and radiological data were retrospectively analyzed. The predictive performance of RAR was evaluated and compared with established clinical scoring systems, including bedside index for severity in acute pancreatitis (BISAP), systemic inflammatory response syndrome (SIRS), harmless acute pancreatitis score (HAPS), and pancreatitis activity scoring system (PASS). *Results*: The median RDW-to-albumin ratio (RAR) was 3.9 (range: 2.6–36.7). Receiver operating characteristic (ROC) curve analysis demonstrated that RAR showed good predictive performance for ICU admission (Area Under the Curve (AUC): 0.781; *p* < 0.001; optimal cut-off: 4.15) and high predictive performance for in-hospital mortality (AUC: 0.927; *p* < 0.001; optimal cut-off: 5.26). RAR exhibited limited but statistically significant discriminatory performance when compared with the BISAP score (AUC: 0.591; *p* = 0.017), whereas no significant predictive performance was observed in relation to PASS, HAPS, or SIRS scores. *Conclusions*: Within the context of this retrospective cohort, RAR is a simple, inexpensive, and readily available biomarker that may be associated with ICU admission and in-hospital mortality in patients with AP. Given the absence of standard severity endpoints such as persistent organ failure or pancreatic necrosis, these findings should not be interpreted as evidence of conventional disease severity prediction but rather as hypothesis-generating observations that warrant validation in larger prospective studies.

## 1. Introduction

AP is an inflammatory disorder that can manifest across a broad clinical spectrum, ranging from mild, self-limiting inflammation to severe systemic involvement that may progress to multiple organ dysfunction and death. Early identification of patients at risk for adverse clinical outcomes is critical for evaluating prognosis and planning timely and effective treatment strategies [1].

Although several scoring systems, such as Ranson, Acute Physiology and Chronic Health Evaluation II, BISAP, HAPS, and SIRS, have been developed to assess disease severity or clinical risk, these models often have limited practical applicability because of their complexity, time-consuming nature, and reliance on multiple parameters [2]. Therefore, there is an increasing need for easily applicable, low-cost, and rapid biomarkers to support early risk stratification in AP.

In recent years, hematological parameters such as RDW and serum albumin have emerged as independent prognostic indicators owing to their strong association with systemic inflammation and nutritional status [3]. RDW reflects erythrocyte size variability and serves as an indirect indicator of the systemic inflammatory response, whereas serum albumin, a marker of hypoproteinemia, tends to decrease during inflammatory states and has been associated with poor clinical outcomes [4,5].

The combination of these two parameters, expressed as RAR, has been proposed as an integrated marker reflecting both inflammatory burden and physiological reserve. Its prognostic value has been demonstrated in various clinical settings, including sepsis, pneumonia, and coronavirus disease 2019 (COVID-19) [6,7,8].

Recent studies in AP suggest that RAR may be associated with clinically important outcomes, including mortality and the need for intensive care. In a retrospective study based on the MIMIC-IV database involving 499 ICU patients, Xuan Chen et al. reported that an RAR value > 5.14 was significantly associated with both short- and long-term mortality, with ROC analysis showing comparable or even superior predictive performance compared with the standard SOFA score [9]. Similarly, in a prospective study by Acehan et al. involving 365 patients, an RAR value > 4.35 measured at 48 h after admission was associated with an increased risk of developing a severe clinical course, and the AUC for predicting mortality was reported as 0.960 [10].

Accordingly, the present study aimed to assess the prognostic significance of the red cell distribution width-to-albumin ratio in AP by examining its association with intensive care unit admission, in-hospital mortality, and commonly used clinical severity scoring systems, rather than formal severity classification according to the Revised Atlanta criteria, in order to evaluate its potential utility in clinical decision-making.

**Figure 1:**
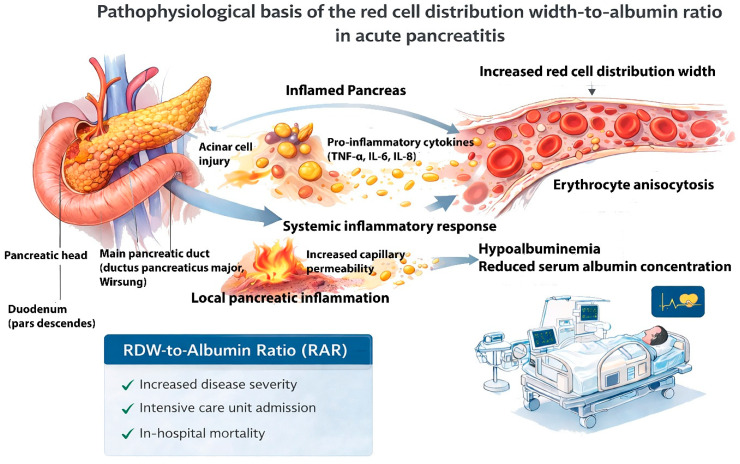
Pathophysiological basis of the red cell distribution width-to-albumin ratio in acute pancreatitis. Acinar cell injury in AP triggers local pancreatic inflammation and the release of pro-inflammatory cytokines (TNF-α, IL-6, IL-8), leading to a systemic inflammatory response and increased capillary permeability. These processes result in erythrocyte anisocytosis, reflected by increased red cell distribution width, and hypoalbuminemia due to reduced serum albumin concentration. The combined effect of elevated RDW and decreased albumin leads to an increased RDW-to-albumin ratio, which is hypothesized to be associated with adverse clinical outcomes, including ICU admission and in-hospital mortality, rather than formal disease severity as defined by the Revised Atlanta Classification.

From a pathophysiological perspective, RDW reflects systemic inflammation and alterations in erythrocyte homeostasis, whereas serum albumin represents nutritional status, hepatic synthetic function, and vascular permeability. RAR, as a composite marker, integrates these complementary processes and may therefore provide a concise reflection of both inflammatory burden and physiological reserve in AP. The underlying mechanisms contributing to this association are illustrated in Figure 1.

## 2. Materials and Methods

### 2.1. Study Design and Population

This retrospective observational study included patients diagnosed with AP who were hospitalized and followed up at Mersin University Hospital between January 2019 and February 2024. Clinical data were retrospectively analyzed.

The diagnosis of AP was established according to the Revised Atlanta Classification, requiring the presence of at least two of the following three criteria: (i) abdominal pain consistent with AP, (ii) serum amylase and/or lipase levels exceeding three times the upper limit of normal, and (iii) characteristic imaging findings on abdominal ultrasonography or computed tomography. Although the diagnosis of acute pancreatitis was based on the Revised Atlanta Classification, this study did not aim to classify disease severity according to Atlanta-defined categories. Accordingly, detailed categorization into mild, moderately severe, and severe acute pancreatitis, as well as systematic assessment of persistent organ failure and pancreatic necrosis, was not consistently available for all patients due to the retrospective nature of the study and variability in imaging and clinical documentation.

**Figure 2:**
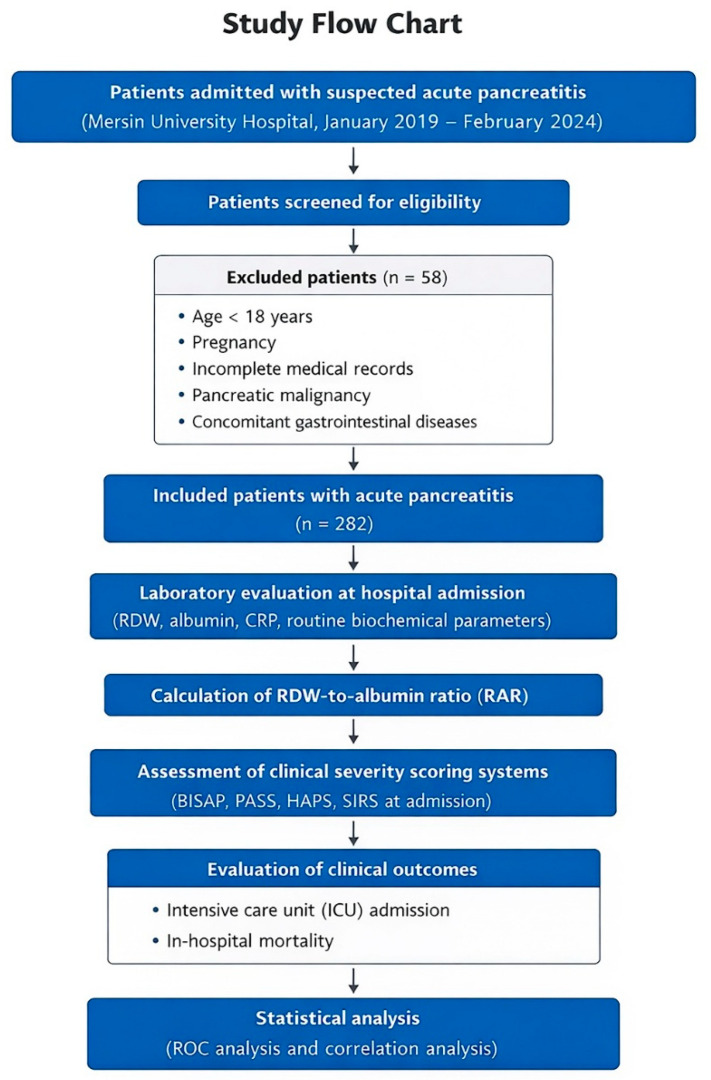
Study flow chart. Flow chart illustrating patient selection, exclusion criteria, and study design. Patients admitted with suspected AP at Mersin University Hospital between January 2019 and February 2024 were retrospectively screened. After exclusion of patients who did not meet the eligibility criteria, a total of 282 patients with AP were included in the final analysis. Laboratory parameters obtained at hospital admission were used to calculate RAR. RAR was analyzed in relation to clinically relevant outcomes, including ICU admission and in-hospital mortality, as well as its association with commonly used clinical severity scoring systems (BISAP, PASS, HAPS, and SIRS).

Patients aged 18 years or older who met the diagnostic criteria and had complete clinical and laboratory data available at the time of hospital admission were eligible for inclusion. Exclusion criteria were the presence of benign or malignant pancreatic tumors, incomplete or missing medical records, age under 18 years or pregnancy, and coexisting gastrointestinal diseases. After applying these criteria, a total of 282 patients were included in the final analysis. The patient selection process and study design are summarized in Figure 2.

### 2.2. Data Collection

Demographic data, including age, sex, BMI, and etiology, as well as comorbidities such as hypertension, diabetes mellitus, and hyperlipidemia, were collected from patient medical records. Information on lifestyle factors, including a history of alcohol consumption and smoking, was also recorded. Laboratory parameters, comprising complete blood count, biochemical analyses, C-reactive protein (CRP), RDW, and serum albumin levels, were obtained for all patients.

RAR was calculated by dividing RDW (%) by the serum albumin concentration (g/dL). Serum albumin values at hospital admission were available for 232 of the 282 patients; therefore, the RDW-to-albumin ratio could be calculated only for patients with available albumin measurements. Analyses involving RAR were performed in this subgroup. In addition, data regarding the type of hospital admission (general ward or ICU), clinically relevant outcomes, including intensive care unit admission and in-hospital mortality, and commonly used clinical severity scoring systems (BISAP, HAPS, PASS, and SIRS) were recorded. Standard severity endpoints defined by the Revised Atlanta Classification, such as persistent organ failure and pancreatic necrosis, were not systematically collected and were therefore not included as outcome variables. All laboratory parameters were derived from blood samples collected within the first 24 h of hospital admission. The exact interval between symptom onset and blood sampling could not be reliably determined due to the retrospective design.

### 2.3. Ethics Approval and Consent to Participate

This study was conducted in accordance with the ethical principles outlined in the Declaration of Helsinki. Owing to the retrospective nature of the study, informed consent was not obtained from the participants. Ethical approval was granted by the Clinical Research Ethics Committee of Mersin University Faculty of Medicine (approval date: 9 July 2025; approval number: 766), which also approved a waiver of the informed consent requirement.

### 2.4. Statistical Analysis

Statistical analyses were performed using SPSS software version 22.0 (IBM Corp., Armonk, NY, USA). The normality of data distribution was assessed using the Kolmogorov–Smirnov and Shapiro–Wilk tests. Variables that did not follow a normal distribution are presented as median (minimum–maximum). To assess the potential impact of missing albumin data, a sensitivity analysis was performed by comparing baseline demographic characteristics and major clinical outcomes between patients with available albumin measurements and those without. Continuous variables were compared using appropriate non-parametric tests, and categorical variables were compared using chi-square or Fisher’s exact test, as appropriate.

Given the exploratory nature of this study and its focus on early risk stratification using a simple admission-based biomarker, multivariable regression analyses were not performed. The predictive performance of RAR for clinically relevant outcomes, including in-hospital mortality and ICU admission, and its association with commonly used clinical severity scoring systems was evaluated using ROC curve analysis. Formal disease severity according to the Revised Atlanta Classification was not analyzed as an outcome. For ROC-derived cutoff values that achieved statistical significance, sensitivity, specificity, positive predictive value (PPV), and negative predictive value (NPV) were calculated.

The association between RAR and clinical severity scoring systems was assessed using Spearman’s rank correlation coefficient. A *p* value < 0.05 was considered statistically significant.

## 3. Results

### 3.1. Baseline Demographic and Clinical Characteristics

The study included 282 patients with a mean age of 55.29 ± 17.93 years. The median body mass index (BMI) was 28.5 (21.0–59.6), and the median number of AP attacks was 1 (0–10). The gender distribution was 54.3% female and 45.7% male (Table 1).

In sensitivity analyses, patients with available serum albumin measurements (n = 232) and those without available albumin data (n = 50) showed no significant differences in age, sex distribution, ICU admission, or in-hospital mortality.

### 3.2. Etiological Distribution

Biliary pancreatitis was the most common etiological factor, accounting for 61.7% of cases, followed by hypertriglyceridemia (16.0%). Other etiological causes are summarized in Table 2.

### 3.3. Laboratory Findings

Baseline laboratory characteristics of patients with AP are presented in Table 3. The RDW-to-albumin ratio (RAR) was available for a total of 232 patients and had a median value of 3.9 (range: 2.6–36.7).

### 3.4. Clinical Characteristics and Outcomes

Clinical characteristics, comorbidities, ICU admission, plasmapheresis requirement, and in-hospital mortality are summarized in Table 4. Because standard disease severity endpoints defined by the Revised Atlanta Classification, including persistent organ failure and pancreatic necrosis, were not consistently available across the cohort, they were not included as outcome variables in the present analyses.

### 3.5. Predictive Performance of RAR

The median RDW-to-albumin ratio (RAR) was 3.9 (range: 2.6–36.7) (Table 3). ROC curve analysis was performed to evaluate the predictive value of RAR for clinically relevant outcomes, including ICU admission and in-hospital mortality. When statistically significant cutoff values were identified, sensitivity, specificity, PPV, and NPV were calculated (Table 5).

**Figure 3:**
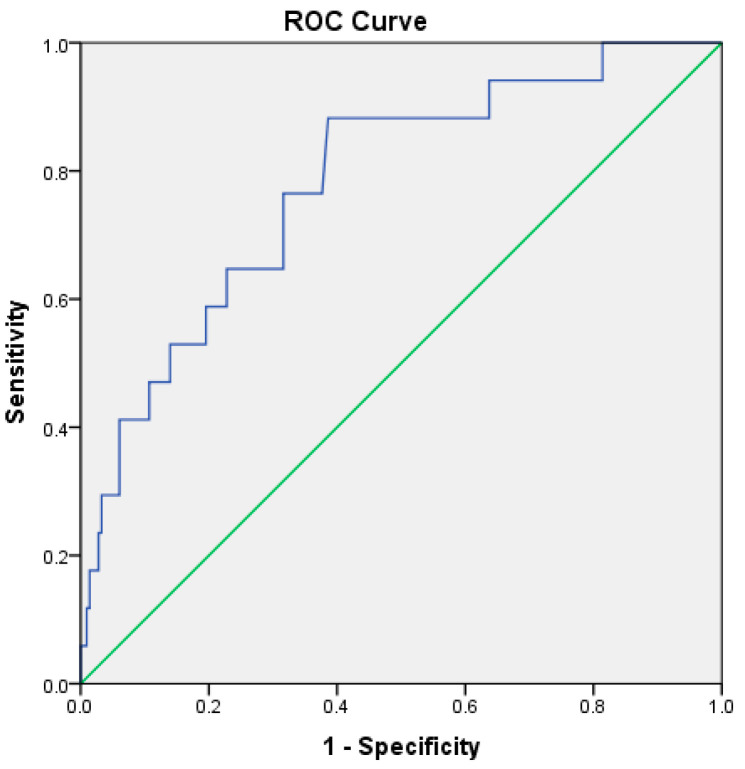
ROC curve illustrating the discriminatory performance of the RDW-to-albumin ratio for ICU admission in patients with acute pancreatitis within the present cohort. The blue line represents the ROC curve of the model, while the green diagonal line indicates the reference line corresponding to no-discrimination.

**Figure 4:**
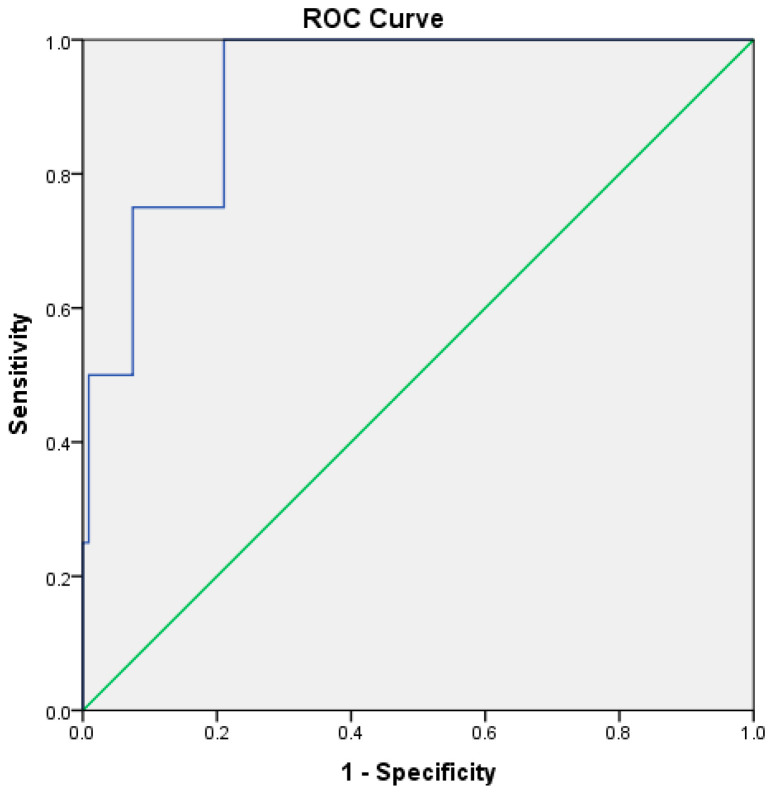
ROC curve illustrating the discriminatory performance of the RDW-to-albumin ratio for in-hospital mortality in patients with acute pancreatitis within the present cohort. The blue line represents the ROC curve of the model, while the green diagonal line indicates the reference line corresponding to no-discrimination.

RAR demonstrated good predictive performance for ICU admission, with an AUC of 0.781 (*p* < 0.001). The optimal cutoff value was 4.15, yielding a sensitivity of 76% and a specificity of 68% (Table 5, Figure 3). For in-hospital mortality, RAR demonstrated high prognostic discrimination within this cohort, with an AUC of 0.927 (95% CI: 0.83–1.00, *p* < 0.001) (Table 5, Figure 4).

For predicting in-hospital mortality, RAR showed high prognostic discrimination within this cohort, with an AUC of 0.927 (*p* < 0.001). The optimal cutoff value was 5.26, corresponding to a sensitivity of 75% and a specificity of 92% (Table 5, Figure 4).

### 3.6. Comparison of RAR with Clinical Severity Scores

**Figure 5:**
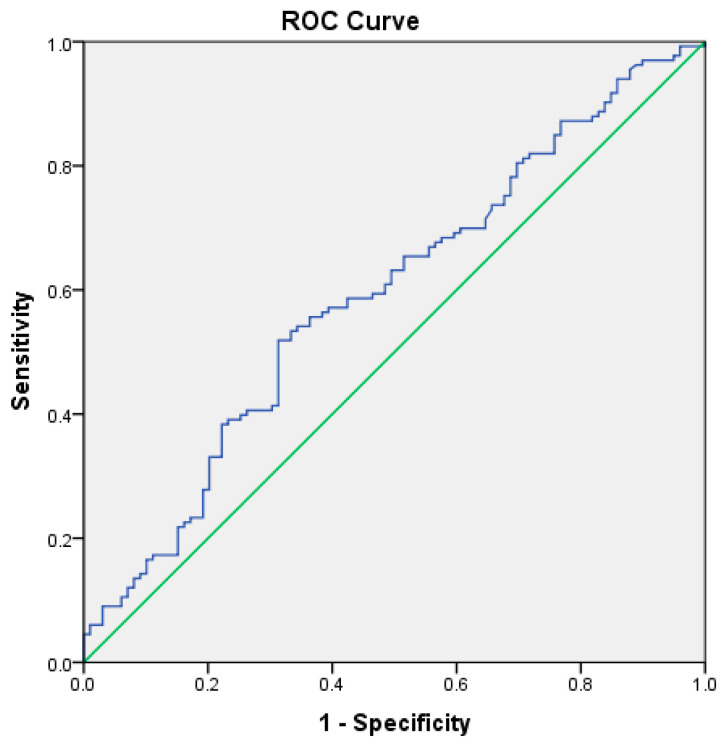
ROC curve illustrating the exploratory discriminatory overlap between the RDW-to-albumin ratio and the BISAP score, used for contextual comparison rather than formal performance evaluation, in patients with acute pancreatitis. The blue line represents the ROC curve of the RDW-to-albumin ratio, while the green diagonal line indicates the reference line corresponding to no-discrimination.

ROC analysis was also performed to explore the discriminatory association between RAR and commonly used clinical severity scoring systems, rather than formal disease severity outcomes. For the BISAP score, the AUC was 0.591 (*p* = 0.017), indicating limited but statistically significant overlap in risk stratification (Table 5, Figure 5).

In contrast, no statistically significant discriminatory overlap was observed between RAR and PASS (*p* = 0.207), HAPS (*p* = 0.061), or SIRS (*p* = 0.221) scores (Table 5).

### 3.7. Correlation Between RAR and Clinical Scoring Systems

The distribution of RAR, PASS, HAPS, and SIRS scores was assessed using histograms, probability plots, and the Kolmogorov–Smirnov and Shapiro–Wilk tests. As these variables did not follow a normal distribution, Spearman’s rank correlation analysis was applied.

No statistically significant correlations were found between RAR and PASS (*p* = 0.23), HAPS (*p* = 0.08), or SIRS (*p* = 0.26), suggesting that RAR may provide complementary prognostic information related to clinical outcomes, rather than directly overlapping with these clinical severity scoring systems.

## 4. Discussion

The results of this study indicate that RAR may be associated with clinically important outcomes in patients with AP. Notably, RAR demonstrated a high discriminatory performance within this retrospective cohort for predicting both in-hospital mortality and the need for intensive care. In our analysis, the AUC for mortality prediction was 0.927, and a cutoff value of 5.26 yielded a sensitivity of 75% and a specificity of 92%. However, given the very small number of mortality events, these estimates should be interpreted with caution, as ROC-based performance measures derived from few events are prone to statistical instability and optimism. Accordingly, these findings suggest that RAR may represent a hypothesis-generating prognostic marker, rather than a parameter that can currently support definitive clinical decision-making.

Similarly, RAR showed a moderate to good discriminatory performance within this cohort for predicting ICU admission, with an AUC of 0.781. A cutoff value of 4.15 corresponded to a sensitivity of 76% and a specificity of 68%. These findings suggest that RAR may have potential value for early risk stratification, particularly in settings where rapid assessment is required; however, its role in admission planning or resource allocation should be considered exploratory pending further validation. Several studies in the literature support the prognostic role of RAR in AP. In a prospective observational study by Acehan et al., patients with an RAR value greater than 4.35 at 48 h had an approximately 18-fold increased risk of developing a severe clinical course, and the AUC for mortality prediction reached 0.960 [10]. Similarly, Wang et al. demonstrated that an RAR value exceeding 0.36 (%/g/dL), as defined using their specific unit convention, was associated with more severe disease manifestations, with a sensitivity of over 80% [11].

It should be noted that positive and negative predictive values are inherently dependent on outcome prevalence. Given the relatively low rates of ICU admission (7.1%) and in-hospital mortality (1.4%) in the present cohort, PPVs are expected to be modest even for markers with good discriminatory performance. Accordingly, these measures may be particularly sensitive to random variation in small-event settings. Therefore, PPV and NPV should be interpreted with caution, in the context of prevalence, and viewed as complementary rather than definitive measures of clinical utility, especially in exploratory or hypothesis-generating analyses, such as early triage settings.

Because laboratory measurements were obtained within the first 24 h of hospital admission, variations in symptom onset–to–sampling time and early fluid resuscitation may have influenced RDW and serum albumin levels through hemodilution and acute inflammatory shifts. Accordingly, the proposed RAR cut-off values should be interpreted with caution as cohort-specific, early admission–based thresholds, rather than fixed biological constants or universally applicable clinical decision limits. In addition, direct comparison of RAR cutoff values across different studies should be approached with caution due to potential differences in unit definitions and calculation methods. RDW is commonly reported as a percentage (approximately 12–16%), whereas some studies appear to have used fractional RDW values (e.g., 0.12–0.16) or alternative unit conventions when deriving the RDW-to-albumin ratio. Such methodological differences can result in numerically disparate cutoff values that are not directly comparable unless units are standardized. This issue, which is well recognized in the interpretation of composite seromarker indices, underscores the need to contextualize RAR thresholds within each specific study rather than extrapolating absolute cutoff values across cohorts.

Comparable findings have also been reported in large database analyses, particularly among critically ill populations. In a study conducted by Chen et al. using the MIMIC-IV database, an RAR value greater than 5.14 in ICU-admitted patients with AP was significantly associated with both short-term (in-hospital) and long-term (30-, 90-, and 365-day) mortality. In that cohort, the predictive performance of RAR for mortality was reported as a ROC AUC of 0.927 [9]. However, these results were derived from a large ICU-based population with substantially higher event rates and should therefore be interpreted within the context of that specific clinical setting. Beyond AP, RAR has also emerged as a prognostic biomarker in other critical illnesses, such as sepsis. In a study involving 3969 eligible patients with sepsis, elevated RAR was significantly associated with 30- and 90-day mortality and demonstrated predictive performance comparable to the lactate-to-albumin ratio, with an AUC of 0.633 in ROC analysis [12]. These observations suggest that RAR may reflect a general inflammatory–nutritional risk signal across critical illnesses, although its predictive magnitude appears to vary according to disease context and baseline risk.

From a pathophysiological perspective, RDW reflects systemic inflammation and alterations in erythrocyte lifespan, whereas albumin represents nutritional status and hepatic reserve. As a composite marker, the RDW-to-albumin ratio provides a more comprehensive reflection of both the inflammatory response and the patient’s overall physiological condition. This integrative nature may allow RAR to provide complementary prognostic information alongside established prognostic scoring systems such as BISAP, Ranson, and MCTSI, particularly in the early admission setting [11], rather than serving as a replacement for these tools.

Importantly, established prognostic scoring systems, particularly the Ranson score, remain highly relevant in the contemporary assessment of acute pancreatitis. Despite being developed several decades ago, the Ranson score continues to demonstrate prognostic accuracy comparable to that of newer scoring systems, as emphasized in a recent comprehensive review by Ong and Shelat [13]. Notably, this review highlights that the 48 h timeframe required for full Ranson score calculation should be regarded as an inherent strength rather than a limitation, as it allows for the integration of early disease evolution into risk stratification. In light of this evidence, the present study does not seek to challenge or replace well-validated tools such as the Ranson score, nor does it demonstrate superiority or non-inferiority of the RDW-to-albumin ratio. Instead, RAR is explored as a simple, admission-based biomarker that may provide complementary, exploratory information during the early phase of hospitalization, when comprehensive scoring systems have not yet fully matured.

In our study, RAR showed a statistically significant but modest association with the BISAP score, with an AUC of 0.591 (*p* = 0.017) in ROC analysis. The BISAP score incorporates variables such as blood urea nitrogen level, age, altered mental status, the presence of SIRS, and pleural effusion—parameters that do not fully overlap with the inflammatory and physiological domains captured by RAR. Despite its relatively low concordance with BISAP, RAR may provide complementary information in specific clinical contexts, owing to its simplicity, cost-effectiveness, and rapid availability [14]. In contrast, no statistically significant correlations were observed between RAR and the PASS, HAPS, or SIRS scores in our analysis. These findings suggest that RAR captures a different prognostic dimension than these scoring systems, rather than directly mirroring established severity constructs. Because RDW and serum albumin are influenced by age, comorbidities, nutritional status, renal and hepatic function, and acute inflammatory and fluid shifts, the independent prognostic contribution of RAR beyond established clinical and laboratory variables could not be reliably determined within the scope of this exploratory study.

Beyond these findings, several large-scale observational studies, prospective cohorts, and meta-analyses provide important context for interpreting the prognostic relevance of RAR within the broader landscape of inflammatory and nutritional biomarkers used in acute pancreatitis. Growing evidence suggests that RAR may function as a useful composite inflammatory–nutritional marker for early risk stratification in acute pancreatitis. In the present study, RAR demonstrated a high discriminatory performance within this retrospective cohort for both ICU requirement and in-hospital mortality, although these observations should be interpreted cautiously given the limited number of events. In particular, our mortality AUC value of 0.927 is numerically comparable to the results reported by Chen et al., who analyzed 499 ICU patients with AP from the MIMIC-IV database and demonstrated that RAR values exceeding 5.14 were independently associated with increased short- and long-term mortality across multiple time points. Their restricted cubic spline analysis further revealed that RAR becomes a significant risk factor beyond a clear threshold, supporting the biological plausibility of a dose–response relationship in high-risk ICU populations [9].

Additional evidence supporting the prognostic relevance of RAR in AP has emerged from other large critical care databases and predictive modeling studies. In a retrospective analysis based on the MIMIC-III database, Wu et al. demonstrated that elevated admission RAR was independently associated with increased 28-day all-cause mortality, with an AUC of 0.669 and a cutoff value of 4.39. Importantly, multivariate Cox regression confirmed RAR as an independent predictor of mortality within that large ICU-based cohort after adjustment for multiple clinical and laboratory confounders [15]. Furthermore, Pan et al. incorporated the RDW-to-albumin ratio into a novel prognostic nomogram for predicting 30-day mortality in AP. In that model, RAR emerged as one of the most influential laboratory variables, and the nomogram demonstrated improved predictive performance compared with established scoring systems such as SOFA, OASIS, and APS III, highlighting the potential additive value of RAR when integrated with clinical parameters in multivariable models [16].

The prognostic value of RAR is further reinforced by cumulative evidence from systematic synthesis. In a recent meta-analysis, Hussaini et al. pooled data from five retrospective studies and demonstrated that elevated admission RAR was associated with a more than two-fold increase in mortality risk, as well as more severe clinical outcomes, as defined within the respective included studies. Importantly, this meta-analysis highlights that despite moderate heterogeneity, the direction of effect remained consistent across different clinical settings, suggesting a reproducible association of RAR as a prognostic marker [17]. Taken together, these studies indicate that the prognostic relevance of RDW-, albumin-, and ratio-based biomarkers in acute pancreatitis has been increasingly recognized. In this context, the present study should be viewed as providing supportive, admission-based, and hypothesis-generating evidence for the potential utility of RAR, rather than introducing a fundamentally new prognostic concept. These findings are broadly consistent with our results and further contextualize our observations.

Beyond mortality, RAR appears to be particularly useful for early prediction of a severe clinical course in acute pancreatitis, as suggested by prior studies. Wang et al. demonstrated that an RDW-to-albumin ratio greater than 0.36 effectively discriminated patients with more severe disease manifestations from those with mild disease, with both sensitivity and specificity exceeding 80%, showing performance comparable to established scoring systems such as Ranson, BISAP, and MCTSI [11]. Similarly, Acehan et al., in a prospective cohort study, reported that RAR measured at 48 h after admission was one of the strongest independent predictors of severe disease progression, with an approximately 18-fold increase in risk and an AUC of 0.960 for mortality prediction [10]. Although our study focused on admission values rather than dynamic measurements, and did not formally assess disease severity according to the Revised Atlanta Classification, these findings collectively suggest that RAR may reflect evolving inflammatory burden and physiological reserve during the early course of AP.

The biological rationale underlying RAR’s prognostic value is supported by earlier investigations examining RDW and albumin separately. RDW has long been recognized as a marker of systemic inflammation and impaired erythropoiesis, with multiple studies demonstrating its association with adverse clinical outcomes, including ICU requirement and mortality, in AP [10,18]. Zhang et al. demonstrated that elevated RDW at early admission was significantly associated with a more severe clinical course and ICU requirement, highlighting its role as an early inflammatory marker [19]. Similarly, Li et al. showed that RDW was independently associated with severe disease manifestations and mortality, although its discriminative performance varied when compared with other inflammatory markers such as the neutrophil-to-lymphocyte ratio and prognostic nutritional index [20]. In addition, Cifci and Halhalli reported that RDW, together with albumin and other inflammatory parameters, was significantly associated with mortality and length of hospital stay in patients presenting to the emergency department with AP. These findings support the concept that combining RDW with albumin into a composite index, such as RAR, may yield a more stable prognostic signal than RDW alone [21]. Notably, Zhang et al. showed that RDW is negatively correlated with serum albumin and positively correlated with clinical severity scores, renal dysfunction markers, and ICU admission [10]. Albumin, on the other hand, reflects nutritional status, hepatic synthetic function, and capillary integrity, all of which are compromised during severe systemic inflammation. Albumin-based indices such as the albumin–bilirubin score have been shown to outperform traditional severity scores in predicting in-hospital mortality in critically ill AP patients, lending further support to the prognostic relevance of albumin-centered and ratio-based biomarkers, rather than single-parameter inflammatory indices [22].

Combining RDW and albumin into a single ratio may therefore offer a more comprehensive reflection of both inflammatory burden and physiological reserve. This integrative approach may help explain why RAR may provide more consistent prognostic information than RDW alone or other single-parameter inflammatory markers. Several comparative studies have shown that RDW alone may yield inconsistent results across cohorts, whereas albumin-containing ratios such as RAR or CRP/albumin provide more reliable prognostic information [23,24]. Within the context of the present cohort, our findings are consistent with this concept, as RAR demonstrated a measurable discriminatory performance for ICU admission and in-hospital mortality, without implying superiority over or replacement of established scoring systems.

Importantly, the prognostic significance of RAR is not limited to AP. Large population-based studies have demonstrated that elevated RAR is independently associated with all-cause and cause-specific mortality in the general population, exhibiting a nonlinear dose–response relationship [25]. These observations suggest that RAR may reflect a broader state of systemic vulnerability, rather than a disease-specific phenomenon, which could partly explain its observed prognostic associations in critically ill patients with AP.

Despite these strengths, heterogeneity exists in the literature. For example, Donmez and Ayata reported that although RAR tended to be higher in severe acute biliary pancreatitis, group differences did not always reach statistical significance, likely reflecting limited sample size and etiological restriction. Such findings emphasize that RAR should not be interpreted in isolation but rather as part of a comprehensive clinical assessment. Nonetheless, even studies reporting modest or borderline results acknowledge the potential research and prognostic relevance of RAR and call for larger prospective investigations to better define its role and limitations [26].

Taken together, the present findings, when interpreted alongside available external evidence, indicate that RAR is a simple, inexpensive, and readily available biomarker that may have prognostic relevance in AP. Its association with ICU admission and in-hospital mortality at an early stage suggests a potential role in early risk stratification, although its impact on clinical decision-making should be considered exploratory and requires confirmation in larger, prospective studies, particularly in emergency and resource-limited settings.

**Figure 6:**
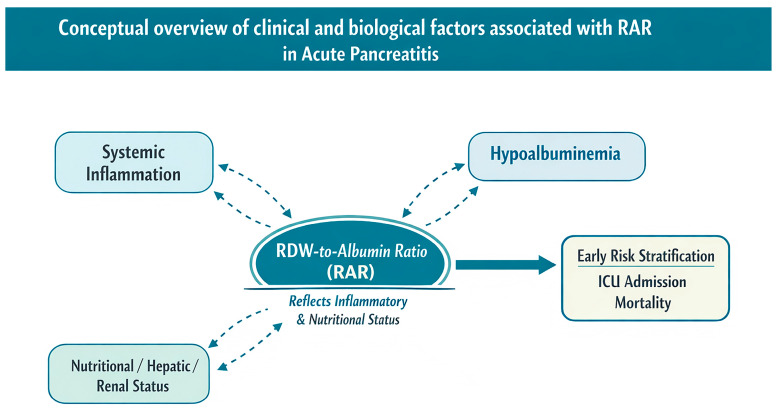
Conceptual schematic illustrating clinical and biological factors associated with RAR in acute pancreatitis. The diagram summarizes potential associations between systemic inflammation, nutritional and organ-related factors, changes in red cell distribution width and serum albumin levels, and adverse clinical outcomes. This schematic is intended as a conceptual framework to aid understanding rather than a definitive mechanistic or causal model and illustrates the hypothesized role of RAR as an admission-based composite marker, without implying a validated or practice-changing application, in early risk stratification.

To facilitate conceptual understanding, the proposed pathophysiological links between systemic inflammation, hypoalbuminemia, erythrocyte anisocytosis, and adverse clinical outcomes are summarized in a schematic overview. This visual representation integrates the biological and clinical rationale underlying the prognostic relevance of the RDW-to-albumin ratio and illustrates its hypothesized association with early risk stratification, rather than implying a definitive or practice-changing role, in AP (Figure 6).

This study has several limitations. First, its single-center and retrospective design may limit the generalizability of the findings and introduce potential selection bias. Second, serum albumin measurements were not available for all patients, which restricted the calculation of the RDW-to-albumin ratio to a subset of the cohort and may have introduced additional selection bias. Third, only laboratory parameters obtained at the time of hospital admission were evaluated, and dynamic changes in RAR during follow-up were not assessed. Therefore, the prognostic value of serial or time-dependent RAR measurements could not be determined. In addition, residual confounding cannot be completely excluded, as factors such as underlying comorbidities, baseline nutritional status, chronic inflammatory conditions, and hepatic function may influence RDW and albumin levels. Renal dysfunction, including chronic kidney disease and acute kidney injury reflected by elevated creatinine levels, may have influenced both ICU requirement and serum albumin concentrations; however, stratified or adjusted analyses according to renal function were beyond the scope of this exploratory study. Furthermore, given the low number of mortality events and the multiple comparisons performed, mortality-related findings should be interpreted with caution as exploratory and hypothesis-generating, with a potential risk of random error and optimistic effect estimates. Moreover, because standard severity endpoints defined by the Revised Atlanta Classification, including persistent organ failure and pancreatic necrosis, could not be systematically assessed due to limitations in retrospective data availability, the present study does not address disease severity in the conventional Atlanta sense. Finally, although RAR was compared with established severity scoring systems, some of these scores are designed to incorporate clinical and laboratory variables over 48–72 h, which may limit direct comparability with admission-based biomarkers. Future multicenter, prospective studies incorporating larger and more diverse patient populations, as well as serial RAR measurements and integrated prognostic models, are warranted to further validate and refine the potential clinical relevance of RAR in AP.

Future research should focus on validating the prognostic value of RAR through large-scale, multicenter, prospective studies that include diverse patient populations and different etiological subtypes of AP. In particular, investigating the temporal dynamics of RAR through serial measurements may provide deeper insight into disease progression and allow for more accurate risk stratification beyond admission-based assessment. Additionally, integrating RAR into composite prognostic models or nomograms alongside clinical variables, inflammatory markers, and established severity scores may help improve predictive performance in research settings and facilitate individualized risk assessment. The potential role of RAR-informed management strategies—such as early intensive monitoring, timely ICU referral, or resource allocation in emergency settings—should be regarded as exploratory and hypothesis-generating, and warrants further investigation before any clinical implementation can be considered. Finally, mechanistic studies exploring the pathophysiological links between erythrocyte anisocytosis, hypoalbuminemia, systemic inflammation, and microcirculatory dysfunction may help clarify the biological basis of RAR and support its potential prognostic relevance, pending further validation, in AP and other critical inflammatory conditions.

## 5. Conclusions

This study suggests that the red cell distribution width-to-albumin ratio may be associated with intensive care unit requirement and in-hospital mortality in patients with acute pancreatitis. However, given the retrospective single-center design and the very small number of mortality events, these findings should be interpreted as hypothesis-generating rather than practice-changing.

In particular, although high AUC values were observed for mortality prediction, ROC-based estimates derived from few events are prone to statistical instability and optimism and should therefore be interpreted with caution. Consequently, the proposed cutoff values should not be regarded as definitive clinical thresholds. Larger, multicenter, prospective studies with adequate event numbers, external validation, and serial measurements are required to determine whether the RDW-to-albumin ratio has a reliable and reproducible role in early risk stratification or clinical decision-making in acute pancreatitis.

## Figures and Tables

**Table 1:** Demographic characteristics of patients with AP.

Variable	n	Value
Age (years)	282	55.3 ± 17.9
Body mass index (kg/m^2^)	255	28.5 (21.0–59.6)
Number of previous AP attacks prior to index admission	282	1 (0–10)

Data are presented as mean ± standard deviation or median (minimum–maximum), as appropriate.

**Table 2:** Etiological distribution of AP.

Etiology	n	%
Biliary	174	61.7
Hypertriglyceridemia	45	16.0
Hypercalcemia	1	0.4
Alcohol-related	9	3.2
Malignancy-related	4	1.4
Drug-induced pancreatitis	3	1.1
Post-ERCP	10	3.5
Idiopathic	25	8.9
Chronic pancreatitis	4	1.4
Pancreas divisum	1	0.4
Intraductal papillary mucinous neoplasm (IPMN)	1	0.4
Autoimmune pancreatitis	1	0.4
Other	4	1.4
**Total**	**282**	**100.0**

**Table 3:** Laboratory characteristics of patients with AP.

Parameter	n	Value
Hemoglobin (g/dL)	282	13.49 ± 2.14
Hematocrit (%)	282	38.74 ± 5.21
White blood cell count (×10^3^/µL)	282	12.15 ± 4.84
Neutrophil count (×10^3^/µL)	282	9.63 ± 4.57
Lymphocyte count (×10^3^/µL)	282	1.63 ± 0.98
Eosinophil count (×10^3^/µL)	282	0.11 ± 0.16
Platelet count (×10^3^/µL)	282	264.30 ± 89.16
Glucose (mg/dL)	278	160.10 ± 77.41
Blood urea nitrogen (mg/dL)	278	18.34 ± 11.50
Aspartate aminotransferase (U/L)	275	182.06 ± 198.53
Alanine aminotransferase (U/L)	276	171.80 ± 194.90
Alkaline phosphatase (U/L)	280	156.62 ± 127.53
Gamma-glutamyl transferase (U/L)	280	254.27 ± 306.23
C-reactive protein (mg/L)	280	46.70 ± 78.61
Total cholesterol (mg/dL)	153	191.44 ± 89.36
Triglycerides (mg/dL)	169	471.76 ± 778.41
Albumin (g/dL)	232	3.55 ± 0.46
Red cell distribution width (%)	282	14.9 (10.5–18.6)
Creatinine (mg/dL)	281	1.1 (0.24–15.3)
Total bilirubin (mg/dL)	281	1.4 (0.05–10.4)
Direct bilirubin (mg/dL)	280	0.4 (0.01–4.8)
RDW-to-albumin ratio (RAR)	232	3.9 (2.6–36.7)

Data are presented as mean ± standard deviation for normally distributed variables and as median (minimum–maximum) for non-normally distributed variables.

**Table 4:** Clinical and demographic characteristics of patients with AP.

Variable	n	%
**Sex**		
Female	153	54.3
Male	129	45.7
**Alcohol use**		
Never drinker	257	91.1
Current drinker	25	8.9
**Smoking status**		
Never smoker	239	84.8
Current smoker	40	14.2
Former smoker	3	1.1
**Hypertension**		
No	181	64.2
Yes	101	35.8
**Diabetes mellitus**		
No	215	76.2
Yes	67	23.8
**Hyperlipidemia**		
No	217	77.0
Yes	54	19.1
Missing data	11	3.9
**Cardiovascular disease**		
No	221	78.4
Yes	61	21.6
**Chronic renal failure**		
No	269	95.4
Yes	13	4.6
**Plasmapheresis**		
No	262	92.9
Yes	20	7.1
**Intensive care unit admission**		
No	262	92.9
Yes	20	7.1
**In-hospital mortality**		
No	278	98.6
Yes	4	1.4

Values are presented as a number (percentage).

**Table 5:** ROC analysis of the RDW-to-albumin ratio for clinical outcomes and its discriminatory performance in relation to established severity scoring systems.

Outcome/Score	AUC (95% CI)	Cut-Off	*p* Value	Sensitivity (%)	Specificity (%)
**Plasmapheresis requirement**	0.45	–	0.592	–	–
**Intensive care unit admission**	0.78 (0.66–0.84)	4.15	<0.001	76	68
**Mortality**	0.93 (0.83–1.00)	5.26	<0.001	75	92
PASS	0.55	–	0.207	–	–
HAPS	0.43	–	0.061	–	–
SIRS	0.55	–	0.221	–	–
BISAP	0.59	–	0.017	–	–

**PASS**: Pancreatitis Activity Scoring System; **HAPS**: Harmless AP Score; **SIRS**: Systemic Inflammatory Response Syndrome; **BISAP**: Bedside Index for Severity in AP.

## Data Availability

The datasets generated and/or analyzed during the current study are available from the corresponding author on reasonable request.

## Abbreviations

AP	Acute Pancreatitis
RDW	Red Cell Distribution Width
ALB	Albumin
RAR	Red Cell Distribution Width-to-Albumin Ratio (RDW/ALB)
ICU	Intensive Care Unit
AUC	Area Under the Curve
ROC	Receiver Operating Characteristic
BISAP	Bedside Index for Severity in Acute Pancreatitis
SIRS	Systemic Inflammatory Response Syndrome
HAPS	Harmless Acute Pancreatitis Score
PASS	Pancreatitis Activity Scoring System
PPV	Positive Predictive Value
NPV	Negative Predictive Value
BMI	Body Mass Index
